# Growth and Osteogenic Differentiation of Discarded Gingiva-Derived Mesenchymal Stem Cells on a Commercial Scaffold

**DOI:** 10.3389/fcell.2020.00292

**Published:** 2020-05-21

**Authors:** Marta Cristaldi, Rodolfo Mauceri, Giuseppina Campisi, Giuseppe Pizzo, Riccardo Alessandro, Laura Tomasello, Maria Pitrone, Giuseppe Pizzolanti, Carla Giordano

**Affiliations:** ^1^Department of Surgical, Oncological and Oral Sciences, University of Palermo, Palermo, Italy; ^2^Department of Biomedical and Dental Sciences and Morphofunctional Imaging, University of Messina, Messina, Italy; ^3^Department of Biomedicine, Neurosciences and Advanced Diagnostics, University of Palermo, Palermo, Italy; ^4^Department of Health Promotion, Mother and Child Care, Internal Medicine and Medical Specialties, University of Palermo, Palermo, Italy

**Keywords:** periodontal disease, bone resorption, waste gingival tissue, oral MSCs, periodontally compromised GMSCs, FISIOGRAFT Bone Granular^®^, Matriderm^®^, autologous bone tissue regeneration

## Abstract

**Background:**

In periodontal patients with jawbone resorption, the autologous bone graft is considered a “gold standard” procedure for the placing of dental prosthesis; however, this procedure is a costly intervention and poses the risk of clinical complications. Thanks to the use of adult mesenchymal stem cells, smart biomaterials, and active biomolecules, regenerative medicine and bone tissue engineering represent a valid alternative to the traditional procedures.

**Aims:**

In the past, mesenchymal stem cells isolated from periodontally compromised gingiva were considered a biological waste and discarded during surgical procedures. This study aims to test the osteoconductive activity of FISIOGRAFT Bone Granular^®^ and Matriderm^®^ collagen scaffolds on mesenchymal stem cells isolated from periodontally compromised gingiva as a low-cost and painless strategy of autologous bone tissue regeneration.

**Materials and Methods:**

We isolated human mesenchymal stem cells from 22 healthy and 26 periodontally compromised gingival biopsy tissues and confirmed the stem cell phenotype by doubling time assay, colony-forming unit assay, and expression of surface and nuclear mesenchymal stem cell markers, respectively by cytofluorimetry and real-time quantitative PCR. Healthy and periodontally compromised gingival mesenchymal stem cells were seeded on FISIOGRAFT Bone Granular^®^ and Matriderm^®^ scaffolds, and *in vitro* cell viability and bone differentiation were then evaluated.

**Results:**

Even though preliminary, the results demonstrate that FISIOGRAFT Bone Granular^®^ is not suitable for *in vitro* growth and osteogenic differentiation of healthy and periodontally compromised mesenchymal stem cells, which, instead, are able to grow, homogeneously distribute, and bone differentiate in the Matriderm^®^ collagen scaffold.

**Conclusion:**

Matriderm^®^ represents a biocompatible scaffold able to support the *in vitro* cell growth and osteodifferentiation ability of gingival mesenchymal stem cells isolated from waste gingiva, and could be employed to develop low-cost and painless strategy of autologous bone tissue regeneration.

## Introduction

Periodontitis is a multifactorial inflammatory disease affecting gingiva and deeper tissues like bone and periodontal ligaments (Löe et al., 1986; Flemmig, 1999; Highfield, 2009). It starts from a localized inflammation of the gingiva, induced by the microorganisms of the dental plaque, that, if not properly treated, progresses until periodontal tissues resorb and create a pocket responsible for tooth loss (Jeffcoat et al., 2003; Gotsman et al., 2007; Darveau, 2010; Lourenço et al., 2014; Feres et al., 2016). Chronicity of periodontitis occurs when >10 of 32 teeth are affected by this pathologic process (Flemmig, 1999; Nair et al., 2014; Julkunen et al., 2018).

Tooth damage or loss is currently treated by replacement with dental implants to restore chewing, speech and esthetic functions (Anusavice, 2012; Ye and Sun, 2017); however, osteointegration is only possible when a sufficient bone volume is available to place the dental implants and establish a strong connection (Brånemark et al., 1977; Albrektsson et al., 1981). It is widely known that patients affected by periodontal disease suffer from bone resorption; in these patients, the restoring of the bone volume necessary to implant dental prosthesis is currently one of the main challenges in dentistry field (Becker and Becker, 1991; Tomasi et al., 2008; Lesolang et al., 2009; Esposito et al., 2014). Thanks to its excellent biocompatibility, osteoinductive and osteoconductive properties, the autologous bone graft represents the “gold standard” of Guided Bone Regeneration (GBR) procedures used to treat bone resorption (Esposito et al., 2009; Kolk et al., 2012). Nevertheless, its use is limited by the requirement of a second surgical site, resulting in an increased probability of clinical complications, higher morbidity and costs of interventions. For these reasons, easier and low-cost dental surgical procedures are urgently needed (Becker and Becker, 1991; Al-Nawas and Schiegnitz, 2014; Esposito et al., 2014).

Regenerative Medicine (RM) and Tissue Engineering (TE), also indicated as TERM, provide new strategies to treat diseases and regenerate injured tissues and organs (Ballini et al., 2017; Gomes et al., 2017). They rely on three main elements to regenerate tissues: mesenchymal stem cells (MSCs), provided of clonogenicity self-renewal and multi-differentiation ability, and biomaterials and bioactive molecules (Langer and Vacanti, 1993; Salgado et al., 2004, 2013; Mason and Dunnill, 2008). Autologous MSCs isolated from human adult tissues represent the ideal stem cell population for autografts (Pittenger et al., 1999; Horwitz et al., 2005; Caplan, 2007; Hipp and Atala, 2008; Ullah et al., 2015). Indeed, thanks to their ability to differentiate toward different cell lineages, human MSCs (hMSCs) can regenerate a wide range of adult tissues such as bone, cartilage, skeletal muscles, tendons, neurons, etc. Moreover, their immunomodulatory, trophic, reparative properties and neuronal plasticity have made hMSCs a valuable candidate for regenerative therapy in the case of tumor ablative techniques in cancer patients as well as an encouraging perspective for use in potential strategies of brain repairing in patients with neurodegenerative diseases (Tatullo et al., 2017).

As it is well known, bone marrow (BM), umbilical cord blood and adipose tissue are currently among the most investigated tissues as sources of hMSCs; however, the harvesting methods can be invasive and painful and, especially in the case of BM, the number, the differentiation potential and the maximal life cycle of hMSCs decrease with the age of the subject (Zuk et al., 2002; Kern et al., 2006). More recently, the oro-facial hMSCs [e.g., dental pulp stem cells (DPSCs), stem cells from human exfoliated deciduous teeth (SHEDs) and gingival mesenchymal stem cells (GMSCs)], have shown promising *in vitro* and *in vivo* TERM potential (Gronthos et al., 2000; Miura et al., 2003; Seo et al., 2004; Sonoyama et al., 2008; Zhang et al., 2010; Egusa et al., 2012; Kawashima, 2012; Jones and Klein, 2013). GMSCs, isolated for the first time in 2009 by Zhang et al. (2010), represent a subpopulation of gingival fibroblasts, with well-demonstrated *in vitro* and *in vivo* abilities of self-renewal, multi-lineage differentiation and immunomodulation (Fournier et al., 2010; Tang et al., 2011).

Many properties make GMSCs ideal for TERM procedures:

1. They are easy to isolate and the patient can be submitted to surgical biopsy without worrying about delayed healing; in addition, the vast majority of dissected gingival tissue is usually discarded during routine surgical procedures (Rossmann et al., 1994; Tang et al., 2011);
2. In presence of specific conditions, GMSCs can differentiate toward mature osteoblasts, chondrocytes and adipocytes, expressing the relative cell lineage markers, phenotype and activity (Wang et al., 2011; Treves-Manusevitz et al., 2013; Xu et al., 2014; Fawzy El-Sayed and Dörfer, 2016);
3. GMSCs have a higher proliferation rate and multi-differentiation ability than bone marrow mesenchymal stem cells (BM-MSCs) (Tomar et al., 2010);
4. GMSCs display a stable phenotype, karyotype and normal telomerase activity in long-term cultures (Tomar et al., 2010).

Furthermore, more recently, inflammatory microenvironments, which characterizes various oral pathological conditions, have demonstrated that they are not only capable of altering the properties of hMSCs but also of improving them in some cases. Further, periapical inflamed cysts are a rich source of immature hMSCs with high regeneration abilities (Tatullo et al., 2017) and the inflammation condition characterizing the periodontally affected periodontium positively affects the stem cell properties of GMSCs, displaying a higher rate of proliferation, expression of MSC markers, and ability of multi-lineage differentiation (Tomasello et al., 2017).

Along with hMSCs, biomaterials are one of the main pillars of bone TERM and, as widely demonstrated, their composition, structure, and properties influence cell attachment, growth, and multi-differentiation (Nooeaid et al., 2012; Asti and Gioglio, 2014). Synthetically produced or naturally derived, biomaterials should have optimal properties, with a functional micro-architecture and well-distributed and interconnected pores along the surface to ensure the regeneration of target tissue (Schumann et al., 2009; Zou et al., 2013). Many types of approaches have been investigated to improve the bone regeneration properties of biomaterials. For instance, nanotechnology has been demonstrated to strongly support the development of scaffolding with its enhanced abilities of bone repair, regeneration, and remodeling (Barry et al., 2016). Additionally, combining nanocomposite scaffolds with cell adhesion and osteoconductive properties, and nanomaterials with osteoinductive and osteoconductive properties may highly improve the bone regeneration ability of a scaffold (Kerativitayanan et al., 2017).

In this study, we confirmed the stem cell phenotype by means of colony-forming unit assay and the expression of the canonical hMSC surface markers (i.e., CD29, CD90, CD73, and CD105) and nuclear markers (i.e., Oct4, SOX2, and NANOG). Afterward, we assessed the *in vitro* cell growth and osteogenic differentiation ability of adult hMSCs derived from waste-inflamed gingiva of periodontal patients on two different types of biomaterials: FISIOGRAFT Bone Granular^®^, a synthetic scaffold consisting of nanohydroxyapatite (NHA) micro granules that is able to mimic the natural bone inorganic phase, and, for this reason, is promising for bone regeneration purposes; and Matriderm^®^, a three-dimensional matrix scaffold consisting of collagen type I (bovine collagen) and elastin (extracted from bovine ligamentum nuchae) that mimics the most represented organic polymer of bone matrix, collagen type I. Matriderm^®^ supports the crucial steps of tissue regeneration and successfully regenerates skin and cartilage tissues (Stark et al., 2006; Ryssel et al., 2008; Keck et al., 2009); however, to our knowledge no study has yet been performed about its potential in bone regeneration.

This study aims to develop a new, easy, and low-cost strategy of autologous bone tissue regeneration by identifying the most suitable scaffold for the growth and osteogenic differentiation of hMSCs derived from discarded gingiva and evaluating the potential of the pro-osteoblastic isoflavone Biochanin A to improve the osteogenic differentiation.

## Materials and Methods

### Ethics

The protocol was approved by the Internal Ethical Committee of the University Hospital A.U.O.P “P. Giaccone” of Palermo (Internal registry: 5/2014). All subjects gave written informed consent in accordance with the Declaration of Helsinki.

### Patient Identification and Gingival Tissue Extraction

Twenty-two healthy adult patients (ages 18–75) who needed their wisdom teeth extracted for orthodontic reasons (control group) and 26 adult patients (ages 18–75) who needed extraction as a result of severe periodontal disease (mobility grade III) (test group), were recruited for the study (females were not suspected to be or visibly pregnant). Both the control and test gingival tissues were resected from gingiva flaps during oral surgery procedures.

Before the extraction each patient was asked to do a mouth rinse with 0.2% chlorhexidine for 1 min (Meridol^®^, Gaba Vebas S.r.l., Rome, Italy) to ensure optimal decontamination of the oral cavity.

### Sample Collection and Establishment of Gingival Cell Cultures

After surgery, the harvested gingival tissues were collected in a 50-ml tube with cold, sterile Dulbecco’s Phosphate Buffer Saline Solution w/o Calcium w/o Magnesium (DPBS w/o Ca^2+^/Mg^2+^) (Euroclone, Milan, Italy), containing 0.25 mg/ml Levofloxacin, 0.40 mg/ml Gentamicin, 5 mg/ml Meropenem, and 0.25 mg/ml Fluconazole, and were transported to the laboratory within 30 min and digested within 3 h.

First, the tissues were mechanically digested using sterile scalpels and then enzymatically digested using a solution of Collagenase Type II (Gibco, Milan, Italy) 1 mg/ml for 2 h at 37°C under agitation. After the digests containing gingival primary cells were centrifuged at 1,200 rpm for 6′, the supernatant was removed, the pellet was re-suspended in fresh Dulbecco’s Modified Eagle Medium/Nutrient Mixture F-12 (DMEM F-12) (Thermo Fisher Scientific, Milan, Italy) containing 10% of fetal bovine serum (FBS) (Euroclone, Milan, Italy), 100 μg/ml Levofloxacin, 50 μg/ml Gentamicin, 50 μg/ml Meropenem, and 1.5 μg/ml Fluconazole, transferred in a T25 culture flask (EuroClone, Milan, Italy), referred to as passage 0 (P0), and incubated at 37°C and 5% CO_2_. The primary cells started to adhere to the flask in 4–5 days and when they resulted in 80% confluence (approximately 2 weeks), they were sub-cultured and referred to as P1. By subculture P3, the antibiotic and antifungal cover had decreased, and by subculture P4 it had completely been abolished. Gingival primary cells between P1 and P6 were used for the experiments in this study.

MSCs isolated from the gingiva of human healthy patients are referred to as H-GMSCs; MSCs derived from gingiva of periodontal disease patients are referred to as P-GMSCs.

### Colony-Forming Unit (CFU) Assay

H-GMSCs and P-GMSCs (P1) were seeded in 10-cm dishes at a density of 300 cells/dish and cultured under conventional conditions; old medium was replaced every 3 days. After 14 days, the cells were washed twice with DPBS, fixed in 4% paraformaldehyde, and stained with 0.1% crystal violet. Cellular groups containing only more than 50 cells were considered colonies. Three sets of experiments for each sample were performed for calculations.

### Population Doubling (DT) and Cell Proliferation Curve

The proliferation rate of H-GMSC and P-GMSCs was evaluated by trypan blue assay (Sigma-Aldrich, Milan, Italy) following the manufacturer’s instructions. H-GMSCs and P-GMSCs (P2) were seeded at a density of 4 × 10^3^ cells/cm^2^ in a 24-well plate and grown up to 120 h. The cells were counted every 24 h by observation under the optical microscope after being stained with trypan blue. The DT was calculated according to the literature data (Roth, 2006, on the website http://www.doublingtime.com/compute.php). Three sets of experiments for each sample were performed for calculations.

### Flow Cytometric Immunophenotyping

H-GMSCs and P-GMSCs (P5) were harvested and the cell pellet was re-suspended in DPBS w/o Ca^2+^/Mg^2+^ at a concentration of 1 × 10^6^ cells/ml; then, 5 × 10^5^ cells/100 μl of cell suspension was used for every cytofluorimetric test.

Briefly, the H-GMSCs and P-GMSCs were tested for expression of hematopoietic stem cell surface markers using FITC human anti-HLA-DR and anti-CD45 monoclonal antibodies and for expression of MSC surface markers using FITC human anti-CD29, CD90, CD105, and PE human anti-CD73 (Table 1). Table 1 describes the conditions of antibody dilution, incubation, and detection, in accordance with the manufacturer’s instructions.

**TABLE 1:** Human anti-monoclonal antibodies list used in flow cytometry analysis for mesenchymal stem cell markers detection.

**Fluorescently-conjugated antibody/localization marker**	**Brand/code number**	**Dilution**	**Incubation**
CD-105/FITC, surface	Milteny Biotec, 130-098-774	1:11	30′, +4°C
CD-29/FITC, surface	Milteny Biotec, 130-101-256	1:11	30′, +4°C
CD-90/FITC, surface	Milteny Biotec, 130-114-859	1:50	30′, +4°C
CD-73/PE, surface PE	Milteny Biotec, 130-111-908	1:50	30′, +4°C
CD-45/FITC, surface	Milteny Biotec, 130-110-631	1:50	30′, +4°C
HLA-DR/FITC, surface	BD Pharmingen, 555811	1:5	30′, +4°C

All reactions were then acquired using the FACS Calibur flow cytometer (Becton-Dickinson, New Jersey, Franklin Lakes, United States) and analyzed by the CellQuest Pro software. Specific IgG isotype antibodies were used as internal negative control. Unstained cells were used as negative control and BM-MSCs as a positive control (not shown).

### Isolation of Total RNA and Real-Time Quantitative PCR (RT-qPCR)

Isolation and purification of total RNA was performed using the RNeasy Mini Kit (Qiagen, Milan, Italy) according to the manufacturer’s instructions. RNA quantity and quality were evaluated by Nano Drop 2000 (Thermo Scientific, Milan, Italy); 2 μg of MSC total RNA were reverse-transcribed to cDNA in a volume of 20 μl with Oligo dT primers using the QuantiTect Reverse Transcription Kit (Qiagen, Milan, Italy). To analyze the stem gene profile and the osteogenic differentiation, quantitative PCR (qPCR) was performed using the QuantiNova SYBR Green PCR Kit and the RotorGene Q Instrument (Qiagen, Milan, Italy). Briefly, the cDNA samples were mixed with SYBR Green PCR master mix and the specific pair of primers is presented in Table 2. The qPCR conditions were as follows: denaturation at 95°C for 3 min for 1 cycle, followed by 44 cycles of denaturation at 95°C for 20 s, annealing at 60°C for 30 s, and elongation at 72°C for 60 s. Three technical replicates were performed for every sample. The specificity of the amplified products was determined by melting peak analysis. The relative expression of target genes was calculated using the ΔΔCt method according to the guidelines (Livak and Schmittgen, 2001). β-actin was used as the housekeeping gene to normalize the expression of target genes, and BM-MSCs—used as a positive cell control—were used to compare gene expression. The results were presented in histograms using GraphPad software setting at 1 the gene expression of the positive cell control. P3 hMSCs were used for the RT-qPCR analysis.

**TABLE 2:** Real-time qPCR primer sequence list for amplification of mesenchymal stem cell cDNA.

**Gene**	**Primer sequence**	**Brand/code number**
β-actin	*F:5′-CCACACTGTGCCCATCTACG-3′ R:5′-AGGATCTTCATGAGGTAGTCAGTCAG-3′*	Eurofins Genomics
NANOG		QT01844808
Oct4		QT00210840
SOX2	*F:5′-GGAGACGGAGCTGAAGCCGC-3′ R:5′-GACGCGGTCCGGGCTTGTTTT-3′*	MWG
RUNX2 (Runt-related transcription factor 2)		QT00020517
OPN (Osteopontin)		QT01008798
OCN (Osteocalcin)		QT00232771

*F = forward; R = reverse.*

### Biomaterials

The biomaterials used in the study were FISIOGRAFT Bone Granular^®^ from GHIMAS Spa (Bologna, Italy), comprising sintered nanohydroxyapatite (NHA) microgranules with a diameter between 0.250 and 0.500 mm and obtained by crashing HA porous blocks, which were derived by a specific burn-out process of polyurethane sponge. MatriDerm^®^ from Medskin Solution (Dr. Otto Suwelack Skin and Health Care GmbH, Billerbeck, Germany), which is a three-dimensional matrix consisting of collagen (bovine collagen) and elastin (extracted from bovine ligamentum nuchae), with a porosity approximately of 100 μm, a size corresponding to 1 cm in length, 1 cm in width, and 1 mm in thickness, and obtained by the Advanced CryoSafe^TM^ Method, which is able to preserve and refine the natural features and properties of the biomaterials.

### Cell Seeding

Both types of biomaterials were provided in sterile conditions. They were incubated in culture media for 30 min at 37°C and 5% CO_2_, prior to cell seeding.

For the viability test, 7,400 cells/cm^2^ were seeded in 5 mg of FISIOGRAFT Bone Granular^®^ scaffold (Gürpinar and Onur, 2005), in a low-adhesion 96-well plate to inhibit the attachment of the cells to the bottom of the well and avoid a false-positive. After seeding, they were incubated at 37°C and 5% CO_2_ and the viability of H-GMSCs and P-GMSCs was evaluated after 24, 48, and 72 h by Water Soluble Tetrazolium Salt 1 (WST1).

To perform the viability test on the Matriderm^®^ scaffold, 10,000 cells/cm^2^ were seeded in the scaffolds using a 24-well plate. After cell seeding, the scaffolds were incubated at 37°C and 5% CO_2_ for 5 min without culture medium to promote the cell attachment; then, 1 ml of fresh complete medium was added to each scaffold and kept at 37°C and 5% CO_2_. After 24, 48, and 72 h, 3-(4,5-dimethylthiazol-2-yl)-2,5-diphenyltetrazolium bromide (MTT) assay was performed to evaluate the viability of the cells.

### WST1 Viability Assay

A WST1 viability assay was performed to evaluate the viability of H-GMSCs and P-GMSCs (P3) seeded on FISIOGRAFT Bone Granular^®^. H-GMSCs and P-GMSCs without scaffolds were used as controls.

Briefly, after 3 h of incubation with 2-(4-iodophenyl)-3-(4-nitrophenyl)-5-(2,4-disulfophenyl)-2H-tetrazolium monosodium salt at 37°C and 5% CO_2_, the absorbance of the supernatant was read at 450 nm, using a microplate reader. Three sets of experiments for each sample were performed for calculations.

### MTT Viability Assay

An MTT viability assay was performed to evaluate the viability of H-GMSCs and P-GMSCs (P3) seeded on Matriderm^®^ scaffold. H-GMSCs and P-GMSCs without scaffolds were used as controls.

Briefly, after 4 h of incubation with 3-(4,5-dimethylthiazol-2-yl)-2,5-diphenyltetrazolium bromide salt at 37°C and 5% CO_2_, the absorbance of the supernatant was read at 570 nm, using a microplate reader. Three sets of experiments for each sample were performed for calculations.

### Live/Dead Assay

Live/Dead assay was performed to evaluate the survival of H-GMSCs and P-GMSCs (P5) seeded on Matriderm^®^ scaffold. Briefly, a dye mix of Ethidium Bromide (100 μg/ml) and Acridine Orange (100 μg/ml) in DPBS was used for the staining. After 24, 48, and 72 h, the scaffolds were washed twice with DPBS (100 μl). Every wash was run for 5 min by slight agitation. Live/Dead dye mix (30 μl) was added to each scaffold for 5 min and images were acquired using a Nikon fluorescence microscope (10×) by FITC (green) and TRITC (red) filters. All images acquired with FITC and TRITC channels were overlapped to distinguish respectively live and dead cells.

### DAPI/Actin Green Assay

The confocal microscopy analysis was performed to evaluate the colonization rate of Matriderm^®^ scaffold by H-GMSCs and P-GMSCs (P5) and the distribution of the cells. Briefly, after 2, 7, and 10 days, the scaffolds were fixed with 4% paraformaldehyde in DPBS (300 μl) at room temperature for 15 min. They were washed with DPBS and incubated with 0.1% Triton-X 100 in DPBS (300 μl) at room temperature for 4 min. Finally, they were incubated with 1:1,000 DAPI (Sigma Aldrich, Milan, Italy) in distilled H_2_O (300 μl) at room temperature for 30 min to stain the nuclei, and 2 drops/ml ActinGreen^TM^ 488 ReadyProbes^TM^ Reagent (Thermo Fisher Scientific, Milan, Italy) in DPBS (300 μl) at room temperature for 1 h to stain cellular cytoskeleton.

The scaffolds were analyzed by Nikon A1 confocal microscope and the software ImageJ^1^. The volumetric analysis has been performed by NIS Elements AR software (Nikon).

### *In vitro* GMSC Osteogenic Differentiation on Matriderm^®^ Scaffold

To test the osteogenic differentiation ability of H-GMSCs and P-GMSCs (P3) grown in the Matriderm^®^ scaffold, the cells were grown in 24-well plates to confluence under standard culture conditions and then maintained in homemade osteogenic differentiation medium (ODM) consisting of DMEM F-12 supplemented with 15% FBS, 10 nM dexamethasone (Sigma-Aldrich, Milan, Italy), 10 mM glycerophosphate (Sigma-Aldrich, Milan, Italy), and 0.05 mM ascorbic acid (Sigma-Aldrich, Milan, Italy), with or without the isoflavone Biochanin A at two different concentrations, 300 nM and 1 μM. H-GMSCs and P-GMSCs cultured without scaffolds were used as a control. After 21 days of culture in the ODM, H-GMSCs and P-GMSCs with or without scaffolds were stained with Alizarin Red S (Sigma-Aldrich, Milan, Italy) to detect calcium deposits. Briefly, scaffolds were transferred in a new 24-well plate, and H-GMSCs and P-GMSCs with or without the scaffolds were gently washed with DPBS, fixed with 4% paraformaldehyde solution for 15 min at room temperature, and rinsed twice with distilled H_2_O. Cells were stained with 40 mM Alizarin Red S (pH 4.1) for 30 min at room temperature with gentle shaking, washed with DPBS and observed under a light optical microscope. The images were acquired with a Nikon DS-fi1. Due to the thickness of the scaffolds, only images of control H-GMSCs and P-GMSCs were acquired. The quantification of the calcium deposits in H-GMSCs and P-GMSCs with or without scaffolds was then evaluated by measurement of Alizarin Red S optical density (OD) at 550 nm.

To perform RT-qPCR analysis, the scaffolds were mechanically digested and incubated with RNA lysis RLT buffer (300 μl) (Qiagen, Milan, Italy), and the supernatant used to perform the RNA extraction and RT-qPCR analysis as above described.

### Statistical Analysis

All the experiments of the study were performed in triplicate, and results are reported as means ± SD and compared by the Student’s unpaired two sample *T*-test. *P* ≤ 0.05 was considered statistically significant.

## Results

### Adherent P-GMSCs Show Higher Proliferation Rate Than H-GMSCs

**FIGURE 1:**
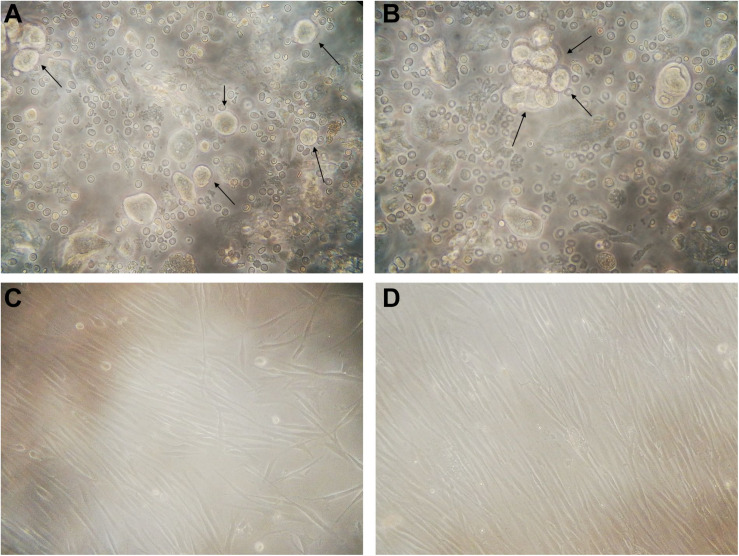
GMSC cultures (P0). Representative image of **(A)** healthy and **(B)** periodontally compromised GMSCs immediately after mechanical and enzymatic digestion, showing a rounded morphology (10×); representative image of **(C)** healthy and **(D)** periodontally compromised GMSCs at 7th day from digestion, with the typical fibroblast-like morphology (10×).

**FIGURE 2:**
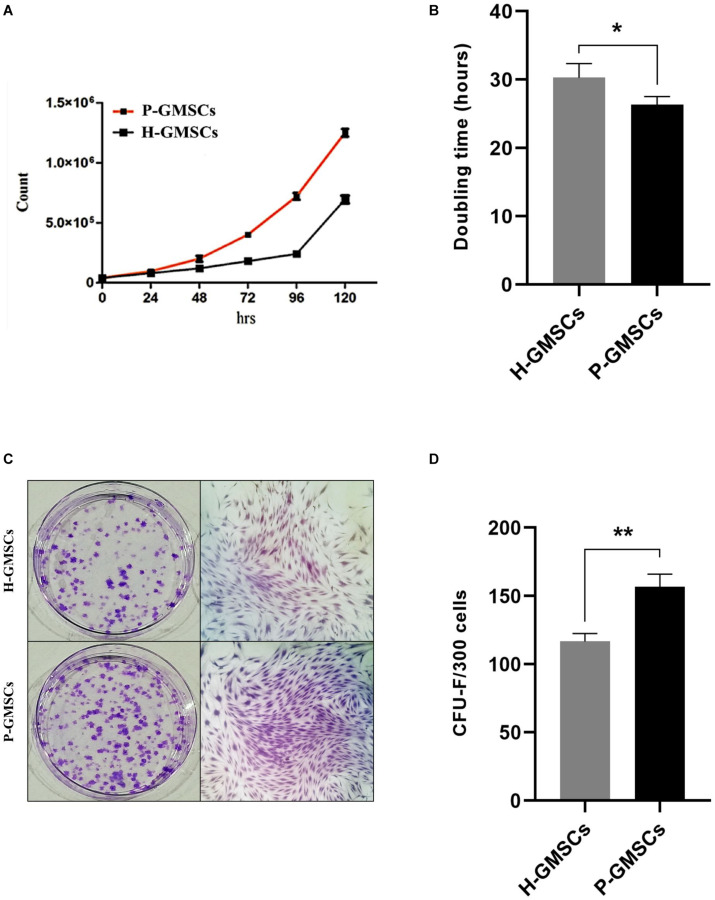
Cell growth analysis and colony-forming unit assay. Panels **(A)** and **(B)** respectively show the cell growth curve of H-GMSCs and P-GMSCs (P2) evaluated by Trypan blue viability assay and the doubling time of H-GMSCs and P-GMSCs calculated according to the literature data (http://www.doublingtime.com/compute.php); Panels **(C)** and **(D)** respectively show the colonies (<50 cells) (left) and the monolayer subculture (right) of H-GMSCs and P-GMSCs (P1) stained with Crystal Violet, and the quantification histogram of the colony-forming unit assay (CFU-F); data are reported as mean values ± SD of three independent experiments. *P*-value *P ≤ 0.05; **P ≤ 0.01.

Twenty-two healthy patients (control group) and twenty-six periodontally affected patients (test group) were used in the study to isolate the GMSCs. For each patient, a gingival flap was used to extract gingival tissue. Ten samples, respectively four of the control group and six of the test group were removed from the study, because of high bacterial contamination. After sequential mechanic and enzymatic digestion, a cell suspension was generated, as shown in the Figures 1A,B, for all 38 samples. Primary cells (P0) derived from both the control and test group started to adhere to the flask approximately between the 4th and the 5th day after digestion. All primary cells from both the control and test group cultures showed a typical fibroblast-like morphology, a homogeneous shape, and size (Figures 1C,D) and reached an 80% confluence between 12 and 18 days; both populations initially showed the same rate of cell growth. After having reached the confluence, they were trypsinized and sub-cultured (referring to them as P1) and then showed a modification in behavior, as is highlighted in cell growth curve (Figure 2A). 24 h after seeding, P-GMSC started to proliferate faster than H-GMSC, showing a higher proliferation rate. The doubling time (DT) was calculated as 26.4 ± 2 h vs. 30.2 ± 1 h (*P* ≤ 0.05), respectively, for P-GMSCs and H-GMSCs (Figure 2B).

### Adherent P-GMSCs Show Increased CFU Ability Than H-GMSCs

To analyze the clonogenic potential of H-GMSCs and P-GMSCs, the CFU assay was performed. GMSCs from both the control and test group were able to form adherent colony-forming units on the plastic dish after 14 days of incubation under standard conditions (Figure 2C); an increase in the number of CFU colonies was observed in P-GMSCs compared to the healthy counterpart, thus showing a higher clonogenic activity. The counting performed by software ImageJ showed 156.8 ± 9.3 and 116.7 ± 5.9 (*P* ≤ 0.01) CFU colonies after 14 days of culture (Figure 2D), respectively, for P-GMSCs and H-GMSCs.

### H-GMSCs and P-GMSCs Are Positive to the Adult MSC Surface and Nuclear Markers and Negative to the Adult Hematopoietic Stem Cell Markers

**FIGURE 3:**
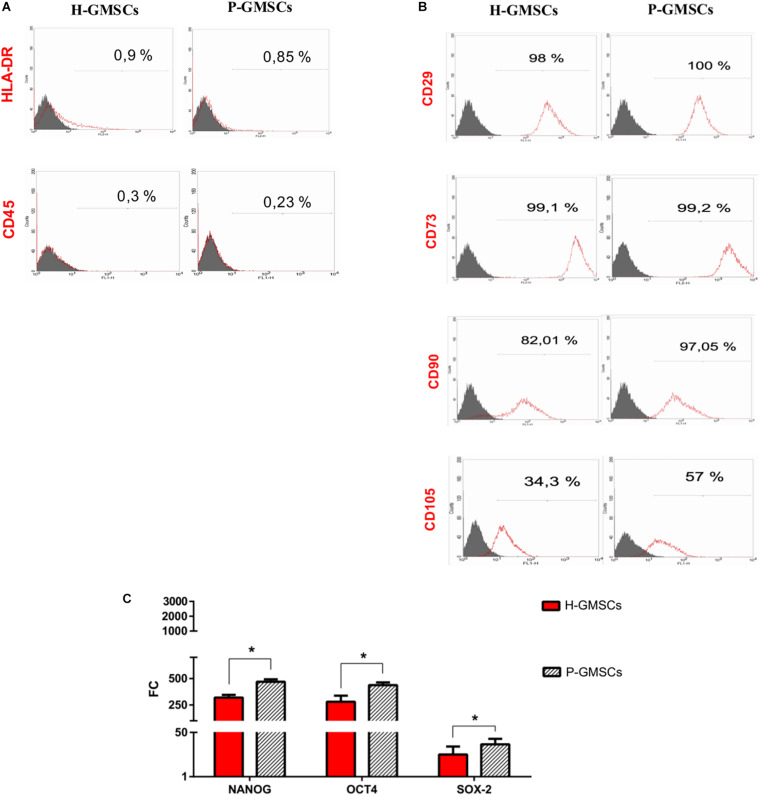
Mesenchymal stem cell feature analysis. Representative fields of flow-cytometric analysis of **(A)** hematopoietic stem cell markers CD45 and HLA-DR and **(B)** MSC markers CD29, CD73, CD90, and CD105 in H-GMSCs and P-GMSCs (P5) (control: isotype anti-IgG1 for CD45, CD29, CD90, CD73, and CD105; isotype anti-IgG2 for HLA-DR); **(C)** the histogram shows the expression of nuclear MSC markers NANOG, Oct4, and SOX-2 in H-GMSCs and P-GMSCs (P3). Data are reported as mean values ± SD of three independent experiments. Actin-β was used as the housekeeping gene; FC = fold change; the mRNA expression of analyzed genes was normalized against BM-MSCs (positive control); *P*-value *P ≤ 0.05.

Both populations of GMSCs analyzed resulted negative for the hematopoietic surface markers CD45 and HLA-DR (Figure 3A) and positive for putative adult MSC surface markers CD73, CD29, CD90, and CD105 (Figure 3B). CD73 and CD29 were highly expressed by all samples (approximately 100%); however, a slightly increased expression of CD90 and CD105 was detected in P-GMSCs compared to H-GMSCs (*P* ≤ 0.05) (Table 3).

**TABLE 3:** Expression levels of MSC markers in healthy and periodontally affected GMSCs.

**MSCs**	**CD-105**	**CD-29**	**CD-90**	**CD-73**
H-GMSCs	34.3 ± 1.2%	98 ± 0.94%	82.01 ± 0.81%	99.1 ± 0.14%
P-GMSCs	57 ± 2.1%	100 ± 0.05%	97.05 ± 0.8%	99.2 ± 0.5%

The expression of adult MSC nuclear markers Oct4, SOX2, and NANOG was positive in both populations, even if it was higher in P-GMSCs than H-GMSCs (*P* ≤ 0.05) (Figure 3C). The adult MSC profile was more highly expressed in P-GMSCs than H-GMSCs.

### WST1 Cell Viability Assay on FISIOGRAFT Bone Granular^®^

**FIGURE 4:**
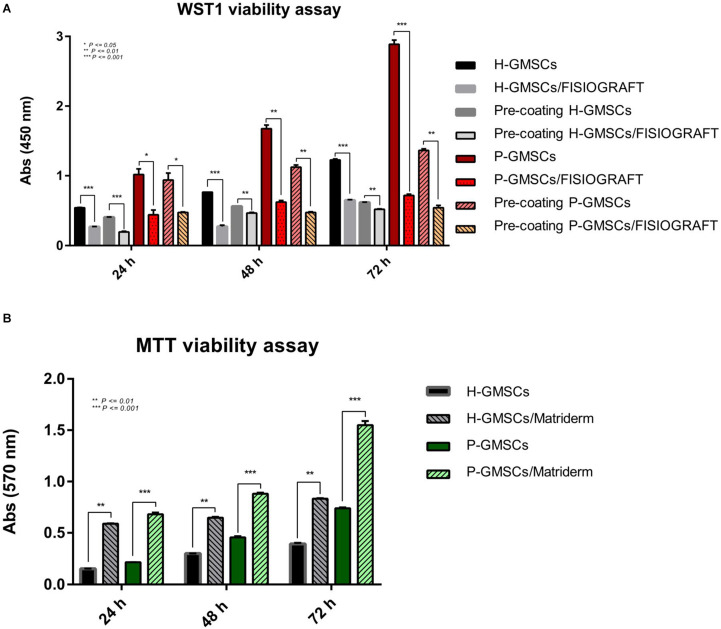
Cell viability analysis. **(A)** WST1 viability values of H-GMSCs and P-GMSCs (P3) grown in the FISIOGRAFT Bone Granular^®^ scaffold for 24, 48, and 72 h; **(B)** MTT viability values of H-GMSCs and P-GMSCs (P3) grown in the Matriderm^®^ collagen scaffold for 24, 48, and 72 h; data are reported as mean values ± SD of three independent experiments; *P*-values *P ≤ 0.05, **P ≤ 0.01, ***P ≤ 0.001.

H-GMSCs and P-GMSCs were seeded on FISIOGRAFT Bone Granular^®^ in the presence of low-adhesion conditions, and the viability evaluated at 24, 48, and 72 h by Water Soluble Tetrazolium Salt 1 (WST1) (Figure 4A). The histogram showed that the viability of H-GMSCs and P-GMSCs grown in presence of the scaffold results in a decrease (roughly 50%) compared to H-GMSCs and P-GMSCs grown without the scaffold, both in standard and low-adhesion conditions, demonstrating that the properties of the scaffold are not suitable for *in vitro* experimental purposes.

### MTT Cell Viability Assay on Matriderm^®^ Collagen Scaffold

H-GMSCs and P-GMSCs were seeded on Matriderm^®^ collagen scaffold for 24, 48, and 72 h and the viability was then evaluated by 3-(4,5-dimethylthiazol-2-yl)-2,5-diphenyltetrazolium bromide (MTT) assay (Figure 4B). The data displayed in the histogram demonstrated the continuous cell growth in presence of the scaffold and a higher proliferation rate of both H-GMSCs and P-GMSCs in presence of the scaffold compared to control cells grown without the scaffold. As expected, the proliferation rate of P-GMSCs was higher than H-GMSCs.

### Live/Dead Assay on Matriderm^®^ Collagen Scaffold

**FIGURE 5:**
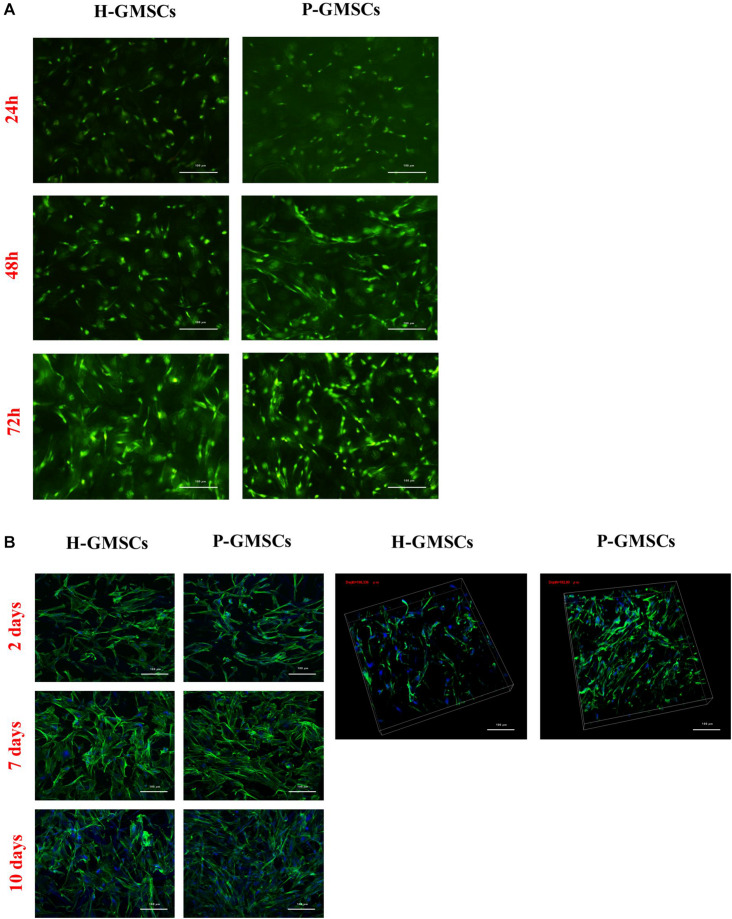
Cell distribution analysis. **(A)** Fluorescent representative images of a Live/Dead assay of H-GMSCs and P-GMSCs (P5) grown for 24, 48, and 72 h in the Matriderm^®^ collagen scaffold (4X); **(B)** (left) MaxI P and (right) volumetric images of DAPI/Actin Green confocal microscopy assay of H-GMSCs and P-GMSCs (P5) grown for 2, 7, and 10 days in the Matriderm^®^ collagen scaffold (4X); scale bars = 100 μm; depth = 190,336 μm for H-GMSCs; depth = 182,80 μm for P-GMSCs.

The viability and the distribution of GMSCs from healthy and periodontally affected tissues in Matriderm^®^ collagen scaffold was also evidenced by the Live/Dead assay (Figure 5A). Approximately 100% of both H-GMSCs and P-GMSCs, seeded in the scaffold for 24, 48, and 72 h, were viable; the density of the cells increased in every time-point and was higher for P-GMSCs than H-GMSCs. We also observed that both H-GMSCs and P-GMSCs tended to align along the direction of collagen fibrils.

### DAPI/Actin Green Assay on Matriderm^®^ Collagen Scaffold

After nuclear and cytoskeleton staining, a confocal microscopy analysis was performed to evaluate the colonization rate of Matriderm^®^ collagen scaffold by H-GMSCs and P-GMSCs after 2, 7 and 10 days of culture under standard conditions. Different areas of the scaffold were taken into consideration and images were acquired. Considering the thickness of the scaffold used (1 mm), the results in Figure 5B showed that both H-GMSCs and P-GMSCs were able to colonize roughly 200 μm of the scaffold and were homogeneously distributed in axes x and y. In particular, H-GMSCs colonized 190,336 μm of the depth of the scaffold and P-GMSCs colonized 182,80 μm of the depth of the scaffold. In addition, we observed an increasing density of cells for up to 10 days, with a higher increase for P-GMSCs compared to H-GMSCs, demonstrating the ability of the cells to colonize the scaffold and grow homogeneously.

### *In vitro* H-GMSC and P-GMSC Osteogenic Differentiation on Matriderm^®^ Collagen Scaffold

**FIGURE 6:**
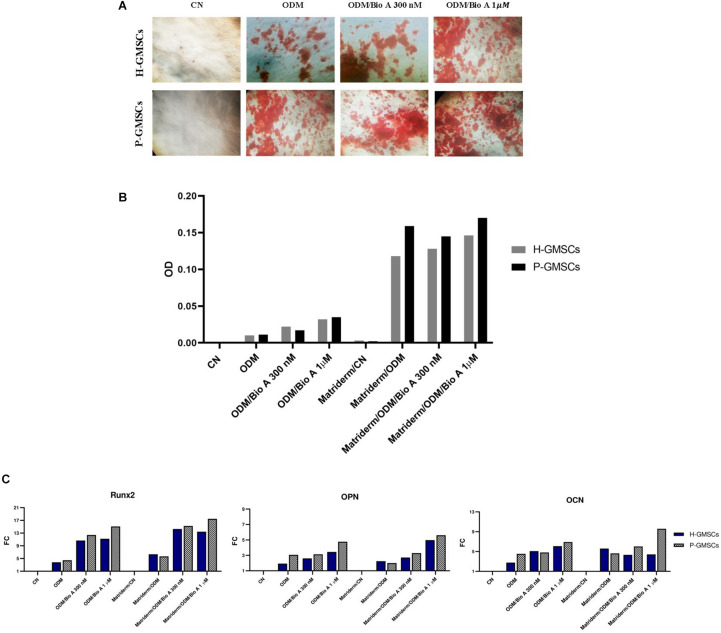
Osteoblastic differentiation assay. **(A)** Representative images of control H-GMSCs and P-GMSCs (P3) grown in osteogenic differentiation medium (ODM), with or without Biochanin A 300 nM and 1 μM, and stained with Red S Alizarin (4×); **(B)** histogram representing the quantitative analysis of Red S Alizarin by spectrophotometry (550 nm OD), of H-GMSCs and P-GMSCs (P3) grown in ODM, in presence or non-presence of the Matriderm^®^ collagen scaffold, with or without Biochanin A 300 nM and 1 μM; **(C)** histogram showing the relative mRNA expression of the osteoblastic markers Runx2, OPN, and OCN in H-GMSCs and P-GMSCs (P3) grown in ODM, in presence or non-presence of the Matriderm^®^ collagen scaffold, with or without Biochanin A 300 nM and 1 μM. Actin-β was used as the housekeeping gene; FC = fold change.

To test the osteogenic differentiation ability of H-GMSCs and P-GMSCs seeded on Matriderm^®^ scaffold, the cells, with or without the scaffold, were grown in 24-well plates to confluence under standard culture conditions and then maintained in homemade ODM, in the presence or non-presence of isoflavone Biochanin A 300 nM and 1 μM and tested by Red S Alizarin assay (Figure 6). After 21 days, the Matriderm^®^ scaffold seemed to support the osteogenic differentiation of H-GMSCs and P-GMSCs with a slight increase in osteoblastic differentiation ability of GMSCs grown in the scaffold; moreover, the presence of Biochanin A at the concentration of 1 μM seemed to induce a slight increase in osteogenic differentiation compared to the standard ODM (Figures 6A,B).

As shown in Figure 6C, the evaluation of the osteoblastic marker expression, i.e., Runt-related transcription factor 2 (Runx2), Osteopontin (OPN), and Osteocalcin (OCN), by RTqPCR analysis, revealed a moderately increased expression in the H-GMSCs and P-GMSCs grown in the Matriderm^®^ scaffold in the presence of Biochanin A, particularly at the concentration of 1 μM, when compared to the GMSCs grown on the plate surface without the scaffold in standard ODM.

## Discussion

Periodontitis is prevalent both in developed and developing countries; it affects around 20–50% of the global population and the high prevalence in young and old people makes it a serious public health concern (Tonetti et al., 2017).

It occurs when the inflammation on gingival tissue, mainly induced by the microorganisms of the dental plaque, is not properly treated and progresses to periodontitis, which is responsible for alveolar bone resorption and tooth loss (Jeffcoat et al., 2003; Gotsman et al., 2007). In these patients, no dental implant can be placed and the physiological oral functions are seriously compromised (Anusavice, 2012; Ye and Sun, 2017). The most frequently applied procedure to treat these types of bone defects is represented by the GBR (Esposito et al., 2009), with autologous bone graft representing the “gold standard.” However, because the autologous bone graft requires a second surgical site, this leads to higher costs of intervention and an increased probability of clinical complications (Esposito et al., 2009; Kolk et al., 2012; Rakhmatia et al., 2013), thus alternative treatments are urgently needed.

TERM is an interdisciplinary field that combines principles of life science, medicine, chemistry, and engineering and has helped to develop many strategies to treat tissue defects (Cristaldi et al., 2018a, b). However, in light of the current sources of hMSCs (i.e., bone marrow, umbilical cord blood, and adipose tissue) (Zuk et al., 2002; Kern et al., 2006), a more accessible and low-cost hMSC source is necessary and the oral cavity seems to be a valuable candidate (Gronthos et al., 2000; Miura et al., 2003; Seo et al., 2004; Sonoyama et al., 2008; Zhang et al., 2010; Gronthos, 2011; Egusa et al., 2012; Kawashima, 2012; Jones and Klein, 2013; Carnevale et al., 2018). Among the different sources of hMSCs identified in the mouth (Miura et al., 2003; Seo et al., 2004; Sonoyama et al., 2008; Zhang et al., 2010; Gronthos, 2011; Egusa et al., 2012; Kawashima, 2012; Carnevale et al., 2018), gingiva seems to be very promising. GMSCs have high regenerative potential, with higher proliferation and multi-lineage differentiation abilities than those of BMMSCs (Rossmann et al., 1994; Tomar et al., 2010; Tang et al., 2011); for these reasons, harvesting hMSCs from the gingiva, and in particular from the gingiva of periodontally compromised teeth that have up to now been discarded during surgical procedures, constitutes an encouraging, easy, and low-cost alternative to the traditional GBR strategies.

In this study, we demonstrated that GMSCs can be isolated both from healthy and periodontally compromised tissues.

Many studies have previously shown that the proinflammatory cytokines such as interleukin (IL)-1β or tumor necrosis factor (TNF)-α can trigger intracellular pathways involved in cellular survival, proliferation, and differentiation toward specific cellular lineages (Hess et al., 2009; Ennis et al., 2013; Feng et al., 2013; Li et al., 2013; Yang et al., 2013; Sun et al., 2014; Fu et al., 2015; Ma and Hottiger, 2016). GMSCs from inflamed tissue acquire a pro-fibrotic phenotype with a higher proliferation rate in the presence of a proinflammatory microenvironment (Li et al., 2013); moreover, TNF-α induces the osteogenic differentiation of DPSCs by activation of the NF-κB pathway (Feng et al., 2013). A role in the inflammation of the Wingless-Type MMTV Integration Site Family, Member 1 (Wnt1)/β-catenin pathway, involved in the transcription activation of stem cell nuclear markers as NANOG, Oct4, and SOX2, has been recently investigated (Ma and Hottiger, 2016). As previously shown by Tomasello et al. (2017), we observed increased clonogenic activity, expression of surface and nuclear MSC markers, and osteogenic differentiation abilities, in GMSCs isolated from inflamed gingiva, confirming that the inflamed microenvironment positively affects the regeneration potential of GMSCs.

To evaluate if P-GMSCs from discarded gingiva could be successfully employed to regenerate the bone, we tested the ability of P-GMSCs and H-GMSCs to grow and osteogenic differentiate in two different types of scaffolds: FISIOGRAFT Bone Granular^®^ and Matriderm^®^. The inorganic phase of bone is mainly constituted of inorganic-based compounds such as hydroxyapatite (HA) (Driessens, 1980), thus HA and calcium phosphate derivates, which mimic the natural bone inorganic phase, have been mostly used for bone regenerative purposes (Li et al., 2002; Lee et al., 2014). The synthetic FISIOGRAFT Bone Granular^®^, provided by Ghimas Spa, consists of granules derived from a HA sponge; it has pores from 500 to 1,000 μm in size and interconnected porosity, which is optimal for cell proliferation. The morphological structure of FISIOGRAFT Bone Granular^®^ mimics the trabecular bone with very thin trabeculae, and it could be a promising scaffold to support the growth and the osteogenic differentiation of GMSCs. A recent clinical study on patients with maxillary sinus bone defect showed that it successfully regenerates the bone defect; 6 months after the FISIOGRAFT Bone Granular^®^ implant in the bone defect, the vital bone percentage was approximately 35%, with marrow spaces percentage of approximately 45%, a residual graft percentage of roughly 21%, and an implant survival rate of 96.4% after 12 months (Stacchi et al., 2017). However, as it is widely accepted, one of the main challenges in the *in vitro* studies is re-producing the *in vivo* cell microenvironment, which is very complex. This is constituted by factors that are able to influence the environment of a cell or a group of cells, with direct or indirect effects on cell behavior and phenotype. A single cell is, indeed, affected by the composition and structure of extracellular matrix (ECM), homotypic and heterotypic cells around the cell, growth factors as cytokines, hormones, and other bioactive molecules with autocrine, endocrine, and paracrine effects; in addition, physical and mechanical factors due to the movement of the organism or the physiological fluids as blood have to be taken in consideration (Barthes et al., 2014). From our results, using the WST1 viability assay, the FISIOGRAFT Bone Granular^®^ does not support the growth of the GMSCs *in vitro* since approximately 50% of both P- and H-GMSCs showed lower viability in the presence of the scaffold. This conclusion may be derived from the marked difference between the *in vitro* and the *in vivo* cellular microenvironment; thanks to the *in vivo* blood supply, many factors can coordinate the biomaterial remodeling and degradation along with the attraction of hMSCs to the target site, supporting tissue regeneration. Additionally, the cells have very different behaviors in 2D and 3D cultures; they, indeed, start to have different behaviors when excised from native three-dimensional (3D) tissues and grow confined to a monolayer. The embryonic stem cells cultured in 3D as embryoid bodies exhibit increased abilities to differentiate to chondrocytes compared to being cultured in the monolayer (Tanaka et al., 2004). The natural cell-cell intercommunication and cell-extracellular matrix interaction in the 3D structure, mimicking the *in vivo* microenvironment, can influence the hDPSC properties and their ability to differentiate toward different cell lineages (Riccio et al., 2010; Xu et al., 2020). Therefore, depending on *in vivo* or *in vitro* microenvironment, cellular intercommunication could be affected and GMSCs could differently respond to the presence of the scaffold.

Type I collagen is the most represented organic polymer of the bone matrix and plays an important role in the complex process of bone formation and remodeling. For these reasons, nowadays, thanks to the excellent biocompatibility, biodegradability, and weak antigenicity, collagen is a biomaterial widely used for tissue regeneration; moreover, the collagen fibrils have demonstrated to serve as a template to guide the bone mineralization (Chvapil and Droegemueller, 1981; Wang and Yeung, 2017). We also tested the growth and osteogenic differentiation properties of P-GMSCs and H-GMSCs on the Matriderm^®^ collagen scaffold, a three-dimensional matrix consisting of collagen type I (bovine collagen) and elastin with a porosity approximately of 100 μm. Our study demonstrated that Matriderm^®^ is able to support the growth of H- and P-GMSCs. The viability results showed a progressively increasing rate of cell growth in the presence of the scaffold. These data were also confirmed by the Live/Dead and DAPI/Actin Green assays, demonstrating that the H- and P-GMSCs, showing the typical fibroblast-like shape, homogeneously colonized the scaffold guided by the collagen fibrils. Besides, we observed an increased cell density up to 10 days of culture. These data proved that the Matriderm^®^ scaffold promoted the adhesion and growth of GMSCs both from the control and test group.

To assess the osteogenic differentiation ability of P-GMSCs and H-GMSCs in the Matriderm^®^ scaffold, H- and P-GMSCs were grown on the scaffold under osteoblastic differentiation conditions. The results derived from Alizarin S Red assay and RTqPCR expression analysis of Runx2, OPN, and OCN show an increased osteogenic differentiation rate of both P-GMSCs and H-GMSCs grown on the scaffold compared to the control cells. However, it was demonstrated that pure collagen materials don’t have enough osteoinductive activity to stimulate bone formation and many strategies, based on scaffold incorporation with bioactive molecules or hMSC treatment with bioactive molecules, have been developed (Otsuka et al., 2013). Biochanin A (5,7-dihydroxy-4′-methoxy-isoflavone), an isoflavone most commonly found in legumes as red clover (Trifolium pratense), which acts as a natural modulator of the estrogen receptor (ER) α and ERβ, is able to enhance the transcriptional pathways physiologically activated by estrogens and inhibited during human pathological conditions, as osteoporosis in postmenopausal women (Joannou et al., 1995; Brynin, 2002; Setchell et al., 2002; Somjen et al., 2005; Yu et al., 2019). In particular, Biochanin A has been showed to enhance the osteoblastic differentiation pathway and inhibit the osteoclastic differentiation pathway (Su et al., 2013a, b), contributing to the maintenance of bone health (Booth et al., 2006; Thompson Coon et al., 2007). Su et al. (2013b) recently demonstrated that Biochanin A at 300 nM contributes to the osteoblastic differentiation; thus, to improve the osteoconductive potential of the Matriderm^®^ scaffold, we evaluated the effects of the isoflavone Biochanin A 300 nM and 1 μM on the osteoblastic differentiation rate of H- and P-GMSCs. Even if only a slight increase in the osteodifferentiation rate of H- and P-GMSCs grown in presence of Biochanin A, in particular at the concentration of 1 μM, was observed, these represent preliminary results and need to be investigated in further studies. Moreover, it is necessary to identify the optimal dose supporting the osteogenic differentiation of P-GMSCs grown in the Matriderm^®^ collagen scaffold. The molecular mechanism, induced by Biochanin A/ERα interaction and involved in the osteogenic differentiation, needs to be also clarified.

## Conclusion

Patients suffering from jawbone loss, as periodontal patients, urgently need a low-cost strategy for alveolar bone defect treatment and regeneration for the placement of dental implants and restoration of oral functions. Bone TE along with RM reproduce tissues and organs by use of adult hMSCs, supported by smart biomaterials and bioactive molecules. In this study, after having confirmed the stem cell phenotype of hMSCs isolated from gingiva of healthy and periodontal patients, we demonstrated that, probably due to the marked difference between the *in vitro* and the *in vivo* cellular microenvironment and behavior, the FISIOGRAFT Bone Granular^®^ does not support the *in vitro* cell growth; future investigations will be necessary to clarify this aspect and improve the system for *in vitro* applications. On the contrary, H-GMSCs and P-GMSCs demonstrated to progressively grow, homogeneously distribute, and osteogenic differentiate in the Matriderm^®^, and the treatment with isoflavone Biochanin A seems to improve the osteodifferentiation rate of the cells. These data need to be confirmed and further investigated in future studies. The optimal concentrations of Biochanin A necessary to support osteogenic differentiation also need to be identified.

Even though preliminary, we believe that hMSCs isolated from waste gingiva, which is routinely discarded during surgical procedures, supported by an osteoconductive scaffold as Matriderm^®^, which is suitable for *in vitro* cell growth and osteodifferentiation, can be employed to develop low-cost and painless clinical strategies of autologous bone tissue regeneration.

Such a system, which uses a biological waste tissue as a source of hMSCs and minimizes the impact on the patient recovery and costs of the surgery, represents an easy and 100% biocompatible alternative to the traditional GBR procedures to treat not only bone defects caused by periodontitis but also any other type of bone defect.

## Data Availability Statement

The datasets generated for this study are available on request to the corresponding author.

## Ethics Statement

The studies involving human participants were reviewed and approved by Internal Ethical Committee of the University Hospital A.U.O.P “P. Giaccone” of Palermo. The patients/participants provided their written informed consent to participate in this study.

## Author Contributions

MC, GC, CG, and RA contributed to the conception and design of the study. MC and GP performed statistical analysis. MC wrote the first draft of the manuscript. All authors contributed to manuscript revision, as well as read and approved the submitted version.

## Conflict of Interest

The authors declare that the research was conducted in the absence of any commercial or financial relationships that could be construed as a potential conflict of interest.

## Footnotes

1 http://imagej.nih.gov/ij/
